# Ki-67 expression as a diagnostic biomarker in odontogenic cysts and tumors: A systematic review and meta-analysis

**DOI:** 10.34172/joddd.2021.012

**Published:** 2021-02-13

**Authors:** Mahnaz Jabbarzadeh, Michael R. Hamblin, Fatemeh Pournaghi-Azar, Maedeh Vakili Saatloo, Maryam Kouhsoltani, Nafiseh Vahed

**Affiliations:** ^1^Department of Oral and Maxillofacial Pathology, Faculty of Dentistry, Tabriz University of Medical Sciences, Tabriz, Iran; ^2^Laser Research Centre, Faculty of Health Science, University of Johannesburg, Doornfontein 2028, South Africa; ^3^Department of Restorative Dentistry, Faculty of Dentistry, Tabriz University of Medical Sciences, Tabriz, Iran; ^4^Department of Oral and Maxillofacial Pathology, Faculty of Dentistry, Urmia University of Medical Sciences, Urmia, Iran; ^5^Research Center for Evidence-based Medicine, Tabriz University of Medical Sciences, Tabriz, Iran

**Keywords:** Biomarker, Ki-67 labeling index, Odontogenic cysts, Odontogenic tumor

## Abstract

Ki-67 is a marker of cell proliferation, used as an important diagnostic marker in the pathologic differentiation of various lesions. It is also relevant for developing targeted molecular therapies. We carried out a systematic review to assess the Ki-67 labeling index (LI) in odontogenic cysts and tumors. Databases were searched, including PubMed (MEDLINE), Scopus, CINHAL, PsycoInfo, the Cochrane Library, and Proquest. The meta-analysis was carried out based on the data of 608 lesions. When a 5% cut-off point was set, ki-67 LI of all benign odontogenic tumors dropped below this point. All the malignant tumors demonstrated an LI of over 15.3%; a significantly higher Ki-67 LI in malignant odontogenic lesions (17.59±2.80) was observed. Among benign tumors, the largest and the smallest Ki-67 LIs were seen in ameloblastoma (4.39±0.47) and adenomatoid odontogenic tumor (0.91±1.71). The mean values of Ki-67 LI in tumors and cysts were 4.23 (0.38) and 1.04 (0.07), respectively. Among odontogenic cysts, the highest Ki-67 LI was found in odontogenic keratocyst (OKC) (3.58±0.51), and the lowest in the radicular cyst (1.29±0.62%). Ki-67 LIs in all odontogenic cysts were <3%, except for OKC. This controversial lesion seems to have a profile more similar to a tumor, and a treatment plan similar to tumors might be suggested. We found that odontogenic lesions have diverse proliferative activities that help differentiate between various lesions and suggest therapeutic plans.

## Introduction

Odontogenic lesions are one of the most common pathologic entities in the jaw region. Odontogenic cysts arise from different types of odontogenic epithelial cell types. The most common odontogenic cyst is the dentigerous cyst, which accounts for 20% of all the mandibular epithelial cysts.^1^ An odontogenic keratocyst (OKC) is classified as a dental lamina cyst with a tendency to relapse after surgery, and it exhibits relatively aggressive clinical behavior. OKCs have a different origin, progression course, and biological behavior from other odontogenic cysts, e.g., dentigerous or radicular cysts.^2^ Relatively high mitotic activity and increased epithelial cell turnover rates have been proposed to account for the growth of OKCs.^3^ In 2005, OKC was classified as a benign odontogenic tumor in the WHO classification; however, it is still debated whether OKC is a benign tumor or an odontogenic cyst.^4^

Ameloblastoma, the most prevalent clinically significant odontogenic tumor, comprises 1% of all jaw cysts or tumors, and 10‒45.2% of all odontogenic tumors. Ameloblastoma is a benign tumor with a tendency for local invasion and a high recurrence rate; it is divided into solid/multicystic, unicystic, and peripheral subtypes.^5^ Unicystic ameloblastoma displays a lining of focal or fully ameloblastic epithelium upon histopathological examination.^6^

Ameloblastic carcinoma is a malignant epithelial odontogenic tumor that might develop within an existing ameloblastoma.^7,8^

Abnormal cell proliferation is an essential feature of tumorigenesis. Ki-67 is a 319–358 kDa protein, considered a reliable marker for the proportion of proliferating cells and to predict the lesion’s behavior.^9,10^ Ki-67 is involved in all the active stages of the cell cycle (G1, S, G2, and M phases), but is not present in the resting G0 phase.^11^ The most common standard for evaluating the tumor for Ki-67 expression is to calculate the percentage of positive nuclear staining with an anti-Ki-67 antibody, which is described as the Ki-67 labeling index (LI).^12^ Ki-67 expression has been used in the diagnosis of many different human tumors, e.g., breast cancer, meningioma, prostate cancer, non-Hodgkin lymphoma, soft tissue sarcoma, etc.^13^

Ki-67 is a diagnostic molecular marker involved in the growth of cancer cells. It is often assayed during the histopathological examination and has also been used for developing therapeutic methods.^14^ In the maxillofacial region, Ki-67 has been used for distinguishing between adenoid cystic carcinoma (ACC) and polymorphous low-grade adenocarcinoma (PLGA), two histopathologically similar lesions, but with different biological behavior and clinical outcome. Based on previous studies, the mean Ki-67 LI for PLGA and ACC has been reported to be 1.24% and 3.71%, respectively.^15^ The Ki-67 LI has also been used to distinguish between two other lesions, basal cell adenocarcinoma (BCAC) and basal cell adenoma. The mean Ki-67 LI values in BCAC and BCA were 4.4% and 2.1%, respectively. The authors suggested a Ki-67 LI cut-off point of 5% and observed that 91% of BCAC LI values were higher than this cut-off point. Accordingly, a high Ki67 LI value could be helpful in BCAC diagnosis.^16^

Ki-67 is a diagnostic and prognostic marker based on many statistical and laboratory findings and is, therefore, a promising candidate to differentiate between various lesions and suggest the best treatment approach.^14^ This study aimed to systematically review the role of Ki-67 LI in odontogenic cysts and tumors. To the best of our knowledge, this is the first review assessing Ki-67 LI in odontogenic lesions.

## Materials and Methods

This systematic review was carried out based on the Preferred Reporting Items for Systematic reviews and Meta-Analyses (PRISMA) guidelines for reporting systematic reviews.

### Search strategies

PubMed (MEDLINE), Scopus, CINHAL, PsycoInfo, the Cochrane Library, and Proquest were used as the search databases.

The key words were selected based on Medical Subject Heading (MeSH) terms. The studies were retrieved by searching for the following keywords:

“odontogenesis”, “odontogenic cyst”, “keratocyst”, “odontogenic keratocyst”, “radicular cyst”, “ki67”, “malignant ameloblastoma”, “ameloblastic sarcoma”, “ameloblastic fibrosarcoma”, “odontogenic carcinoma”, “odontogenic malignancy”, “CEOT”, “KCOT”, “MIB-1 antigen”, “MIB-1 protein”, “antigen MIB 1”, “protein MIB 1”, “Ki67 antigen”, “dentigerous cyst”, “radicular cyst”, “follicular cyst”, “periapical cyst”, “calcifying odontogenic cyst”, “residual cyst”, “glandular odontogenic cyst”, “dental tissue neoplasm”, “odontogenic tumor”, “odontogenic tumor”, “odontogenic adenomatoid tumor”, “odontogenic adenomatoid tumor”, “odontogenic neoplasm”, “odontogenic cancer”, “odontoma”, ”ondontome”, “squamous odontogenic tumor”, “meloblastic sarcoma”, “ameloblastic fibrosarcoma”, “odontogenic sarcoma”, “malignant odontogenic tumor”, “benign odontogenic myxoma”, “ameloblastic fibroma”, “squamous odontogenic tumour” and “calcifying epithelial odontogenic tumor” from 1990 to 2019 (August).

### Study selection and eligibility criteria

The inclusion criteria were as follows:

1. Studies on the Ki-67 expression levels in odontogenic cysts and tumors published from 1990 to 2019 (August).
2. Articles published in the English language with full-text availability.
3. Ki-67 values expressed as a percentage or LI evaluated by counting (the number of positive cells/total number of cells) ×100
4. Ki-67 LI reported as mean ± SD

The following studies were excluded: non-human research, reviews, letters, editorials, expert opinions, articles written in a language other than English, studies that reported ranges instead of means ± SD, studies in which the value was not expressed as a percentage, articles of poor quality, studies that did not include qualitative staining and immunohistochemistry analysis, duplicated articles (risk of bias).

### Quality assessment

Using the CASP (Critical Appraisal Skills Program) quality checklist, the included articles were independently assessed by two specialists in oral and maxillofacial pathology (M.K. and M.V.), and low-quality studies were excluded.

### Data extraction

Two investigators (M.K. and M.J.) independently extracted the articles’ information and summarized them in the standardized extraction form. Extracted data comprised: first author’s name, year of publication, study type, sample size, mean values and standard deviations of Ki-67 expression, lesion type (tumor/cyst), and the lesion name. Endnote X5 Resource Management Software was used to organize titles and abstracts of the articles and identify duplicates.

### Statistical analysis

The Comprehensive Meta-Analysis (CMA; Englewood, NJ, USA) software, version 2.0, was used for data analysis. Mean and standard deviation values of ki-67 expression for each study were extracted from the selected articles. Q statistics and I^2^ were used to determine heterogeneity. A probability value of I^2^ value >50% and *P* value<0.10 for Cochran’s Q indicated significant heterogeneity. Based on heterogeneity analysis, fixed or random-effects models were used. Subgroup analysis was performed based on the type of lesion, the lesion’s name, and the sample size. Statistical significance was set at *P*<0.05.

## Results

### Search results

**Figure 1:**
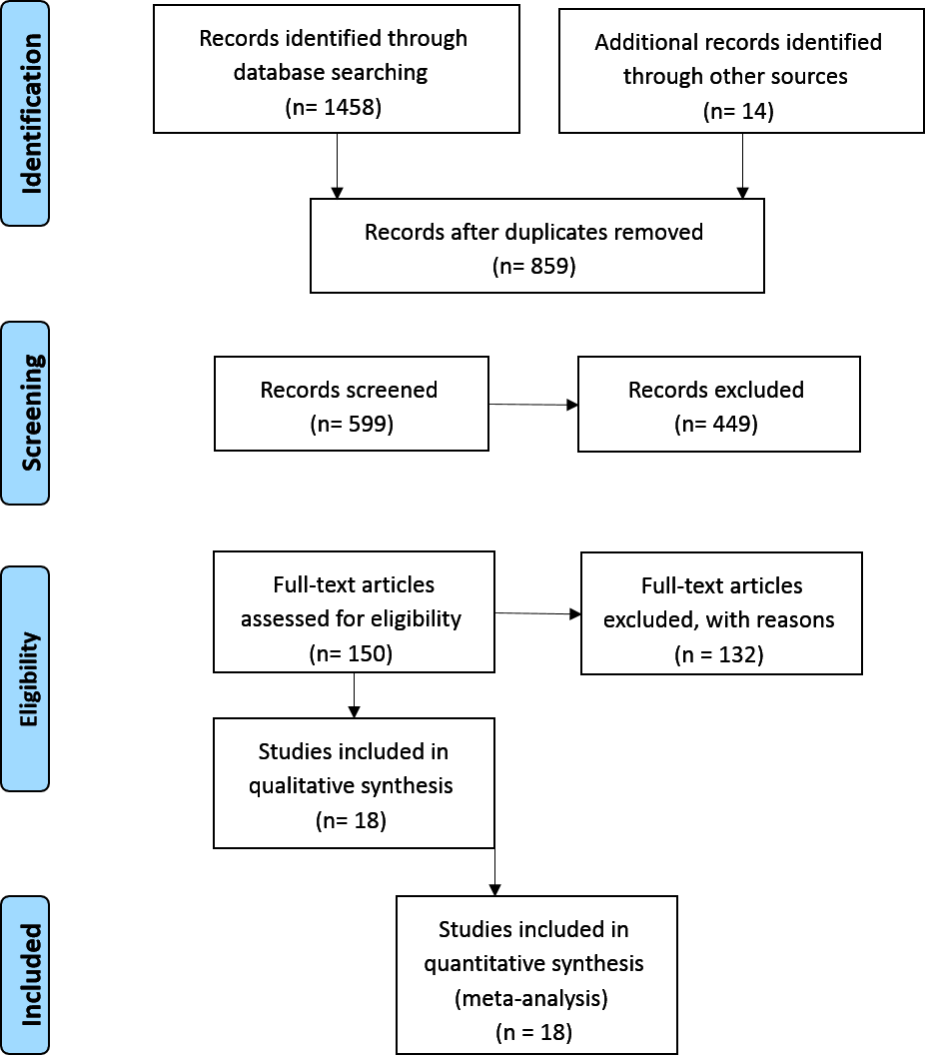
Search strategy and selection process of systematic review.

The initial search retrieved 1458 articles. Of 1458 papers, 859 studies remained to be assessed after the duplicates were removed. A total of 449 articles were excluded after reviewing the titles and abstracts, and the remaining 150 full-text articles were separately reviewed for quality by two investigators. Figure 1 presents the flowchart for the literature search and article selection procedures.

### Characteristics of studies

At this stage, 132 studies were removed because the inclusion and exclusion criteria were not met. A few studies were excluded because of the method they used to report the immunohistochemical results. For example, in one study by Ramadoss et al,^17^ the mean score of Ki-67 was reported instead of Ki-67 LI or percentage. In the study by Otero et al,^18^ the mean ± SD was not reported separately for each lesion, and only a broad comparison was made. Also, in an article by Sathi et al,^19^ the results of immunohistochemical staining were not reported as mean ± SD, and therefore, it was removed from the study. Finally, 18 studies were included in this meta-analysis. Table 1 presents the descriptive characteristics and related data of the included studies.

**Table 1:** Descriptive characteristics and related data from included studies

**Authors**	**Year**	**Country**	**Study type**	**Method**	**Type of lesion**	**No. of samples**	**Samples size**	**Ki-67 LI**
Ong'utL et al^20^	1997	UK	R	IHC	Ameloblastoma (follicular) Ameloblastoma (plexiform)	24 30	5 µm 5 µm	5.0 3.2
Sandra et al^21^	2001	Japan	R	IHC	Ameloblastoma (solid) Ameloblastoma (mixed) Ameloblastoma (desmoplastic) Ameloblastoma (plexiform) Ameloblastoma (follicular) Ameloblastoma (acanthomatous) Unicystic ameloblastoma Unicystic ameloblastoma (cystic type ameloblastoma)	15 8 3 9 9 3 5 9	4 µm 4 µm 4 µm 4 µm 4 µm 4 µm 4 µm 4 µm	5.08 4.24 2.75 5.05 3.70 3.69 2.85 2.88
Yoshida et al^22^	2001	Japan	R	IHC	COC COC Odontoma	16 7 12	3 µm 3 µm 3 µm	2.68 3.73 2.96
Piattelli et al^23^	2002	USA	R	IHC	Dentigerous cyst Unicystic ameloblastoma	8 5	5 µm 5 µm	3.14 5.32
Yanamoto et al^24^	2002	Japan	R	IHC	Ameloblastic carcinoma Ameloblastoma Ameloblastoma (follicular) Ameloblastoma (plexiform)	2 10 7 3	3 µm 3 µm 3 µm 3 µm	12.2 4.2 3.4 5.9
Fregnani et al^32^	2003	USA	R	IHC	COC	10	3 µm	1.3
Suzuki T et al^33^	2005	Japan	R	IHC	Radicular cyst	19	3 µm	1.59
Tsuneki M et al^34^	2008	Japan	R	IHC	OKC Dentigerous cyst Radicular cyst	10 10 10	4 µm 4 µm 4 µm	15.4 0.66 1.4
Razavi et al^35^	2009	Iran	R	IHC	OKC	16	4 µm	6.42
Bello et al^25^	2009	Finland	R	IHC	Ameloblastoma Ameloblastic carcinoma	18 3	5 µm 5 µm	6.4 18.2
Razavi et al^26^	2011	Iran	R	IHC	AOT Ameloblastoma (solid type)	16 16	4 µm 4 µm	0.91 4.3
Ayoub et al^36^	2011	Egypt	R	IHC	OKC Radicular cyst	12 13	4 µm 4 µm	2.68 0.72
Azevedo et al^27^	2013	Brazil	R	IHC	CEOT	19	5 µm	3.04
Olimid et al^28^	2014	Romania	R	IHC	Ameloblastoma (follicular) Ameloblastoma (acanthomatous) Unicystic ameloblastoma (luminal) Unicystic ameloblastoma (intraluminal)	15 2 2 2	4 µm 4 µm 4 µm 4 µm	6.7 4.1 6.2 5.6
Özchamur et al^29^	2014	Turkey	R	IHC	Ameloblastoma Radicular cyst OKC	20 20 20	5 µm 5 µm 5 µm	1.58 1.03 2.84
Živković et al^37^	2017	Serbia	R	IHC	OKC Dentigerous cyst Radicular cyst	30 30 30	4 µm 4 µm 4 µm	0.190 0.260 0.270
Apelláni et al^30^	2018	Uruguay	R	IHC	Ameloblastoma Unicystic ameloblastoma	10 10	5 µm 5 µm	3.04 4.04
Acharya et al^31^	2018	India	R	IHC	Ameloblastoma OKC	30 30	4 µm 4 µm	7.55 8.19

Ki-67 LI: Ki-67labelling index; R: Retrospective; IHC: immunohistochemistry; AOT: adenomatiod odontogenic tumor; CEOT: calcifying epithelial odontogenic Tumor; COC: calcifying odontogenic cyst; OKC: odontogenic keratocyst.

### Publication bias

**Figure 2:**
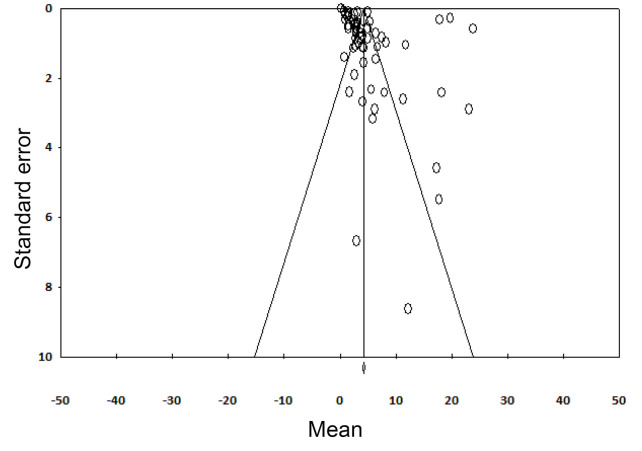
Funnel plot for publication bias test.

An estimation of potential publication bias was carried out using the funnel plot. The asymmetry of the plot suggested publication bias (Figure 2). Egger linear regression test (*P*<0.001) showed statistically significant publication bias.

### Meta-analysis

#### Ki-67 expression in odontogenic tumors

**Figure 3:**
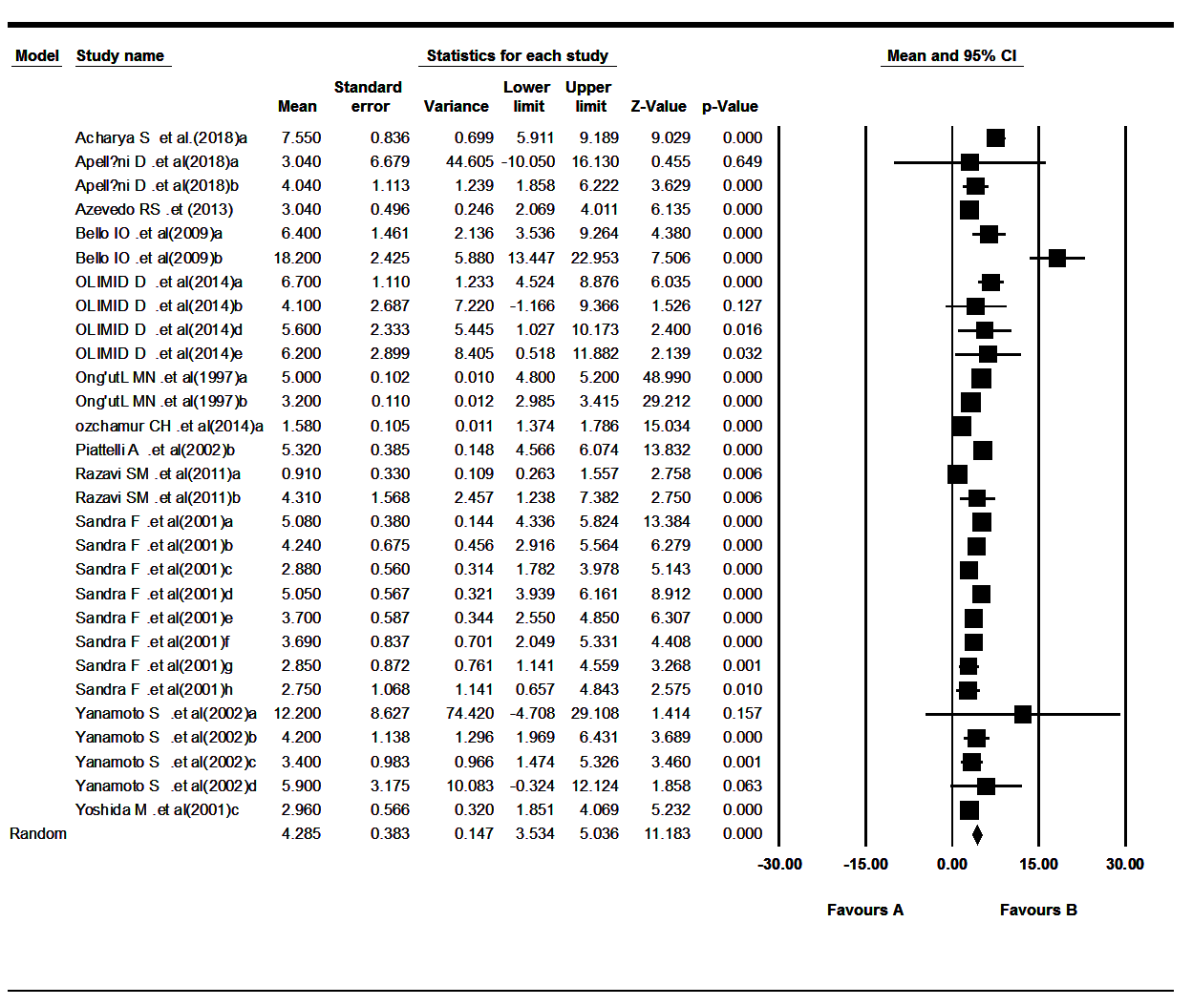
Forest plot diagram of mean Ki-67 expression in odontogenic tumors (study identity).

**Figure 4:**
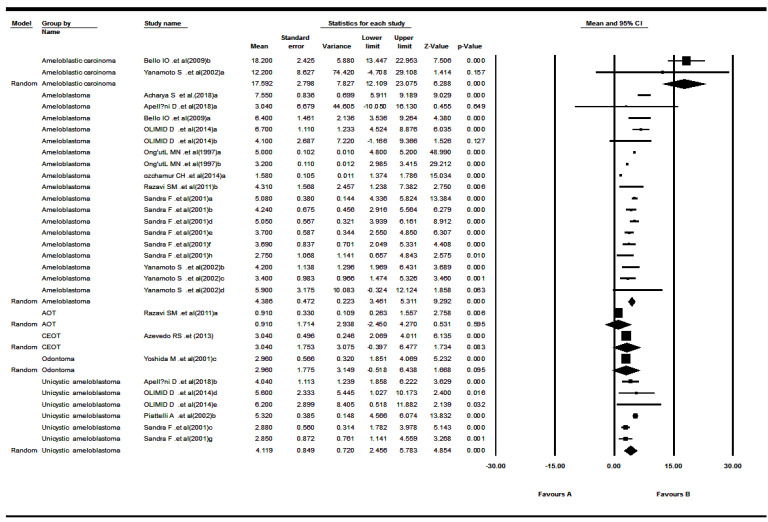
Forest plot diagram of mean Ki-67 expression in odontogenic tumors (type of lesion).

**Figure 5:**
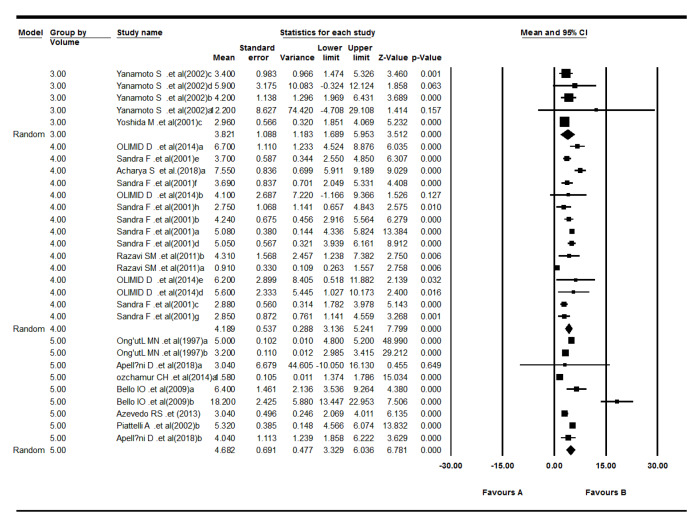
Forest plot diagram of mean Ki-67 expression for biopsy volume in odontogenic tumors.

The 12 included studies reported Ki-67 LI for odontogenic tumors.^20-31^ The results revealed that the mean of Ki-67 expression in tumors was as follows: pooled mean=4.23, SD=0.38 (*P*<0.001). Considering the significant heterogeneity (Q=743.03, df= 28, *P*<0.001, I^2^= 96.23), the random-effects model was used to calculate pooled LI mean at 95% CI (Figures 3 and 4). Subgroup analyses, based on the tumor type and the biopsies’ volume are shown in Tables 2 and 3 and Figure 5, respectively.

**Table 2:** Subgroup meta-analysis results of mean ki-67 expression for tumor type

**Tumor type**	**Effect size and 95% confidence interval**			**Heterogeneity**			
	**Number of studies**	**Point estimate**	**Standard error**	**Q-value**	**df (Q)**	***P*** ** value**	**I** ^2^
Ameloblastic carcinoma	2	17.59	2.80	0.45	1	0.50	0.00
Ameloblastoma	9	4.39	0.47	618.54	17	0.00	97.25
AOT	1	0.91	1.71	0.00	0	1.00	0.00
CEOT	1	3.04	1.75	0.00	0	1.00	0.00
Odontoma	1	2.96	1.77	0.00	0	1.00	0.00
Unicystic ameloblastoma	4	4.12	0.85	16.98	5	0.00	70.56

AOT: Adenomatoid odontogenic tumor; CEOT: Calcifying epithelial odontogenic tumor.

**Table 3:** Subgroup meta-analysis results of the mean ki-67 expression for biopsy volume in tumors

**Biopsy volume**	**Effect size and 95% confidence interval**			**Heterogeneity**			
	**Number of studies**	**Point estimate**	**Standard error**	**Q-value**	**df (Q)**	***P*** ** value**	**I** ^2^
3	2	3.82	1.09	2.73	4	0.60	0.00
4	4	4.19	0.54	123.80	14	0.00	88.69
5	6	4.68	0.69	615.74	8	0.00	98.70

#### Ki- 67 expression in odontogenic cysts

**Figure 6:**
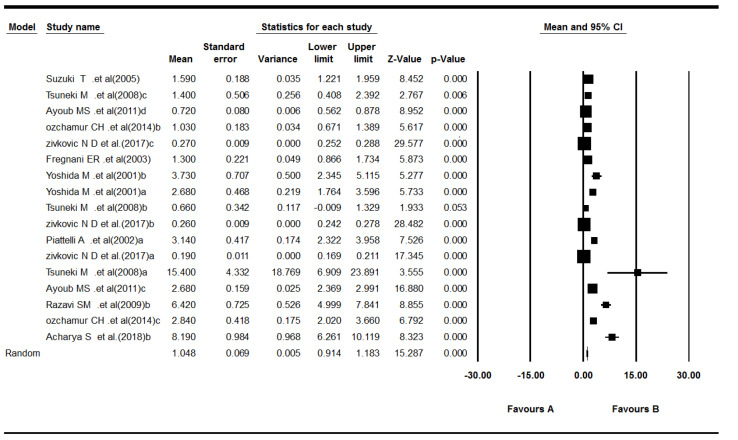
Forest plot diagram of mean Ki-67 expression in odontogenic cysts (study identity).

**Figure 7:**
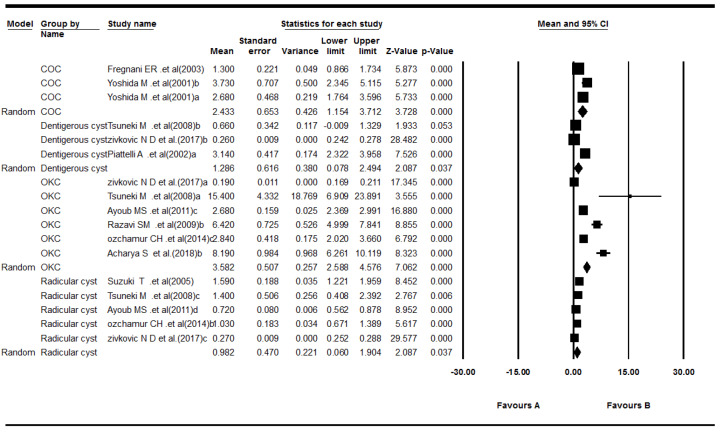
Forest plot diagram of mean Ki-67 expression in odontogenic cysts (type of lesion).

**Figure 8:**
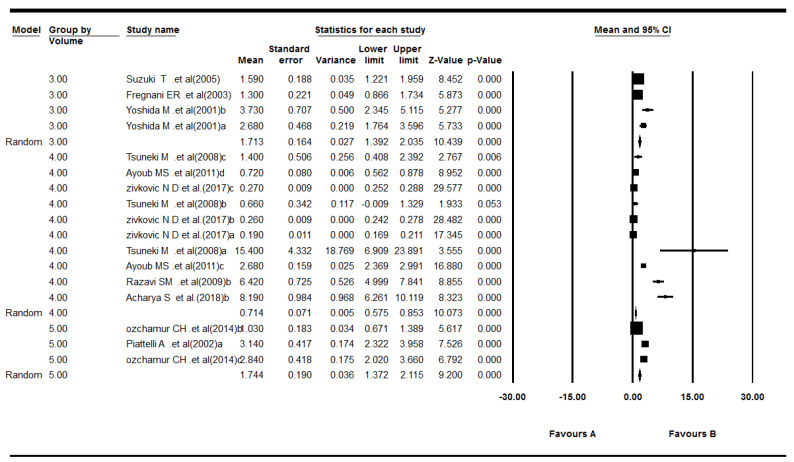
Forest plot diagram of mean Ki-67 expression for biopsy volume in odontogenic cysts.

Ten studies reported Ki-67 expression in odontogenic cysts.^22,23,29,31-37^ The results showed that the mean of Ki-67 expression for cysts was as follows: pooled mean = 1.04, SD = 0.07 (*P*< 0.001). Considering the significant heterogeneity (Q = 688.59, df = 16, *P*< 0.001, I^2^ = 97.67), the random-effects model was used to calculate pooled LI mean at 95% CI (Figures 6 and 7). The results of subgroup analyses, based on the cyst type and the biopsies’ volume, are shown in Tables 4 and 5, and Figure 8, respectively.

**Table 4:** Subgroup meta-analysis results of mean ki-67 expression for cysts type

**Cyst type**	**Effect size and 95% confidence interval**			**Heterogeneity**			
	**Number of studies**	**Point estimate**	**Standard error**	**Q-value**	**df (Q)**	***P*** ** value**	**I** ^2^
COC	2	2.43	0.65	15.90	2	<0.001	87.424
Dentigerous cyst	3	1.29	0.62	48.99	2	<0.001	95.918
OKC	6	3.58	0.51	436.20	5	<0.001	98.854
Radicular cyst	5	0.98	0.47	101.24	4	<0.001	96.049

COC: calcifying odontogenic cyst; OKC: odontogenic keratocyst.

**Table 5:** Subgroup meta-analysis results of mean Ki-67 expression for biopsy volume in cysts

**Biopsy volume**	**Effect size and 95% confidence interval**			**Heterogeneity**			
	**Number of studies**	**Point estimate**	**Standard error**	**Q-value**	**df (Q)**	***P*** ** value**	**I** ^2^
3	3	1.71	0.16	16.12	3	0.001	81.385
4	5	0.71	0.07	460.65	9	<0.001	98.046
5	2	1.74	0.19	32.06	2	<0.001	93.761

## Discussion

The present study was a systematic review to assess Ki-67 LI levels in odontogenic cysts and tumors. The issue’s significance was confirmed by the growth in the number of papers over the period searched. This study’s findings suggested increased Ki-67 LI levels in odontogenic tumors compared to cysts and in malignancies compared to benign lesions.

Although Ki-67 has increasingly been studied as a prognostic indicator in recent years, this marker’s more critical diagnostic role was emphasized in the present study. Simple histopathological techniques are not always able to differentiate between these challenging lesions. Random-effects model analysis was carried out for this purpose. Significantly higher Ki-67 LI levels were noted in ameloblastic carcinoma (17.59 ± 2.80%). The mean value obtained was significantly different from that in benign odontogenic lesions, consistent with previous studies. As observed by Yanamoto et al,^24^ the Ki-67 LI levels in ameloblastic carcinoma (12.2 ± 12.2%) were higher than ameloblastoma (4.2 ± 3.6%). The findings of Bello et al^25^ provide additional support for the statistically significant difference in Ki-67 LI levels between ameloblastoma and ameloblastic carcinoma.

The same crucial diagnostic question has been explored in studies carried out on lesions in other anatomical areas, and the results are comparable to our findings. Faur et al^38^ reported higher Ki-67 expression in the malignant component of carcinoma tissue in pleomorphic adenoma than the benign component. The results were obtained from 29 cases of salivary gland tumors and the Ki-67 LI range for benign tumors was 0.5–6%, but the range was 3.6%–50% for malignant tumors. Therefore, the estimation of the potential proliferation rate of salivary gland tumors and identifying a subgroup of patients with invasive tumors, are useful in diagnostic pathology. This approach can differentiate between benign and malignant lesions and predict the prognosis of salivary gland tumors. In another study by Tadbir et al^39^ on Ki-67 expression in salivary gland tumors, the Ki-67 LI levels in malignant tumors (10.74 ± 10.8%) were higher than benign tumors, such as pleomorphic adenoma (0.78±0.2%).

We also found that Ki-67 LI levels were higher in odontogenic tumors (pooled mean=4.23, SD=0.38, *P*< 0.001) compared with odontogenic cysts (pooled mean=1.04, SD = 0.07, *P*< 0.001). In a similar study, Piattelli et al^23^ found that Ki-67 expression in unicystic ameloblastoma was higher than in dentigerous cysts.

After ameloblastic carcinoma, the highest Ki-67 expression was seen in ameloblastoma (4.39 ± 0.47%), followed by unicystic ameloblastoma (4.12 ± 0.85%). A study by Olimid et al^28^ showed that unicystic ameloblastoma had the lowest proliferation activity compared to other types of ameloblastoma. The Ki-67 LI levels in odontogenic tumors were followed by CEOT (3.04 ± 1.75%), odontoma (2.96 ± 1.77%), and AOT (0.91 ± 1.71%) in descending order. Accordingly, the lowest Ki-67 expression among odontogenic tumors was seen in AOT. These results will be helpful in the pathologic diagnosis and differentiation of these tumors.

As shown in Table 3, there were significant correlations between Ki-67 LI expression and the biopsy volume in odontogenic tumors, except for the volume of 3 µm in odontogenic tumors. These findings might suggest that biopsy volumes of 4‒5 µm would be more appropriate for assessing Ki-67 LI expression in odontogenic tumors.

Among odontogenic cysts, the highest Ki-67 LI expression was seen in OKC (3.58 ± 0.51%), and the lowest was observed in radicular cysts (0.98 ± 0.47%). These findings might be helpful in the histopathological analysis of cysts, especially when inflammation is present.

The order of Ki-67 LI expression in odontogenic cysts was as follows: OKC (3.58±0.51%), COC (2.43±0.65%), dentigerous cysts (1.29±0.62%), and radicular cysts (0.98 ± 0.47%) in descending order. These findings suggest that the assessment of Ki-67 LI in OKC during pathological diagnosis might be useful for diagnostic and therapeutic purposes.^40^ Tsuneki et al^34^ reported that the expression level of Ki-67 in OKC was higher than that in dentigerous and radicular cysts. However, in contrast to our findings, Živković et al^37^ found that Ki-67 expression in OKC was lower than both dentigerous and radicular cysts; this finding might be explained because the evaluation only concerned the suprabasal layer. On the other hand, in a study by Kuroyanagi et al^40^ the expression level of Ki-67 was assessed in both the basal and suprabasal layers, and they observed higher Ki-67 expression in OKC compared with other odontogenic cysts. In another study by Pan et al,^41^ on OKC’s epithelial lining, Ki-67 immunopositive cells were found in both the basal and suprabasal layers.

In the present systematic review, covering 118 cases of OKC, 92 cases of radicular cysts, 33 cases of calcified odontogenic cysts (COC), and 48 cases of dentigerous cysts, we found the highest Ki-67 LI expression in OKC and the lowest expression in radicular cysts (Table 3). Therefore, it can be concluded that Ki-67 is expressed in developmental odontogenic cysts to a greater extent than inflammatory cysts, such as radicular cysts (0.98 ± 0.47%). Among developmental odontogenic cysts, the highest expression of Ki-67 was seen in OKC (3.58 ± 0.51%), with the lowest expression in dentigerous cysts (1.29 ± 0.62%). Selvi et al^42^ reported higher Ki-67 expression in OKC compared with inflammatory and developmental cysts.

According to the data summarized in Table 2 (Ki-67 LI expression in odontogenic tumors), when a cut-off point of 5% is set for Ki-67 LI levels, all the benign tumors drop below this point, while Ki-67 LI for ameloblastic carcinoma is much higher (17.5 ± 2.8%). This fact plays an important diagnostic role and could suggest therapeutic approaches. In Table 4 (Ki-67 LI values in odontogenic cysts), it can be seen that in all odontogenic cysts, Ki-67 LI was less than 3%, except for OKC, which is regarded as a controversial lesion that, according to these findings, seems to be more similar to a tumor than a benign cyst. Although OKC is more commonly classified as an odontogenic cyst, it demonstrates features that distinguish it from other odontogenic cysts.^29^

In the present study, the search strategy was limited to articles published in English. Articles that might contain data with potential high quality, written in other languages, were not included because of difficulties with accurate medical translation. In I^2^ studies with statistical values that were <50%, we used the fixed-effects model and the Mantel-Hansel method, but in other studies where the values were >50% or the P-value was <0.05, the random-effects model was the most reliable option to calculate the overall effect size. The included studies’ heterogeneity might be caused by different methodological techniques, imperfect reporting of cases, absence of true case verification, or other uncertain reasons. Despite these limitations, we believe that crucial diagnostic information can be extracted from the meta‐analysis since the results were based on the best available evidence.

## Conclusion

In this systematic review, we found that when a cut-off point of 5% was set, ki-67 LI of all benign odontogenic tumors dropped below this point. All malignant tumors demonstrated an LI of >15.3%; a significantly higher Ki-67 LI in malignant odontogenic lesions (17.59±2.80) was observed. Among odontogenic tumors, the maximum and the minimum Ki-67 LIs were found in ameloblastoma (4.39±0.47) and AOT (0.91±1.71). Among odontogenic cysts, the highest Ki-67 LI was seen in OKC (3.58±0.51), with the lowest for radicular cysts (1.29±0.62). Ki-67 LI in all odontogenic cysts was <3%, except for OKC. OKC might be more similar to a tumor, and a treatment plan similar to that for tumors might be suggested. Therefore, odontogenic lesions have diverse proliferative activities that help differentiate between various lesions and suggest therapeutic plans.

## Authors’ Contributions

MJ: acquisition of data, drafting the work, revising the paper, and approving final draft. FPA: analysis and interpretation of data, drafting the work, revising the paper, and approving final draft. MVS: acquisition of data, revising the paper, and approving final draft. MK: concept and design of the work, interpretation of data, drafting the work, revising the paper, and approving final draft. MRH: interpretation of data for the work, drafting the work, revising the paper, and approving final draft. NV: acquisition a of data, revising the paper, and approving final draft.

## Funding

This paper is based on a thesis submitted for DDS degree (NO. 62900) and was financially supported by the Research Council of Tabriz University of Medical Sciences.

## Competing Interests

The authors declare no conflict(s) of interest related to the publication of this work.

## Ethics Approval

The research protocol was approved by the Ethics Committee of Tabriz University of Medical Sciences (IR.TBZMED.VCR.REC. 1398.331).
